# Prevalence and correlates of receipt by smokers of general practitioner advice on smoking cessation in England: a cross‐sectional survey of adults

**DOI:** 10.1111/add.15187

**Published:** 2020-07-24

**Authors:** Sarah E. Jackson, Claire Garnett, Jamie Brown

**Affiliations:** ^1^ Department of Behavioural Science and Health University College London London UK

**Keywords:** Brief advice, general practice, GP advice, quit attempts, smoking cessation

## Abstract

**Background and Aims:**

Advice from a general practitioner (GP) can encourage smokers to quit. This study aimed to estimate the prevalence and correlates of receipt of GP advice on smoking, what type of advice and support was offered and characteristics and quitting activity associated with different types of advice.

**Design/setting:**

Data were collected between 2016 and 2019 in a series of monthly cross‐sectional surveys of representative samples of the adult population in England.

**Participants:**

A total of 11 588 past‐year smokers.

**Measurements:**

Participants reported whether they had received advice or offer of support for smoking cessation from their GP in the last year. Socio‐demographic and behavioural characteristics and past‐year quit attempts and cessation were also recorded.

**Findings:**

One in two [47.2%, 95% confidence interval (CI) = 46.1–48.3%] past‐year smokers who reported visiting their GP in the last year recalled receiving advice on smoking, and one in three (30.1%, 95% CI = 29.1–31.1%) reported being offered cessation support. The most common form of support offered was stop smoking services (16.5%, 95% CI = 15.7–17.3%) followed by prescription medication (8.1%, 95% CI = 7.5–8.7%); 3.7% (95% CI = 3.3–4.1%) reported having been recommended to use e‐cigarettes. Smokers who were older, non‐white, more addicted, and smoked five or more cigarettes/day had consistently higher odds of receiving advice or support. There were some differences by region, housing tenure, presence of children in the home and high‐risk drinking in the types of advice/support received. There were no significant differences by sex, occupational social grade, disability, type of cigarettes smoked, or survey year. Advice with any offer of support was associated with higher odds of attempting to quit than advice alone [adjusted odds ratio (OR_adj_) = 1.52, 95% CI = 1.30–1.76]. Advice alone was associated with higher odds of quit attempts than no advice in smokers with higher (OR_adj_ = 1.34, 95% CI = 1.10–1.64) but not lower occupational social grade (OR_adj_ = 0.90, 95% CI = 0.75–1.08).

**Conclusions:**

In England, a minority of smokers receive support from their GP to stop smoking. Those who do are more likely to be older, non‐white and more addicted to cigarettes. Advice plus offer of support appears to be associated with increased odds of making a quit attempt, while advice without offer of support appears only to be associated with increased odds of making a quit attempt in higher occupational social grade smokers.

## Introduction

Tobacco smoking remains one of the leading causes of disease and premature death world‐wide [1]. In England, substantial progress has been made in reducing smoking prevalence over recent decades, but one in seven adults continue to smoke [2]. Brief advice from a general practitioner (GP) can encourage smokers to quit [3, 4], but such advice is not routinely given to all patients [5]. Understanding the extent to which patients are receiving it, what type of advice (and support) is being offered and how far provision of advice (including the type of advice) differs according to patient characteristics, and the possible implications this has for quitting activity is important for the development of effective guidelines and training for health professionals who interact with smokers in primary care.

According to clinical guidelines, GPs in England should assess all patients for smoking annually and advise and encourage every smoking patient to stop smoking [6, 7]. This approach supports the emphasis on prevention in the NHS Long Term Plan [8], which includes increasing the support available to help people to manage and improve their own health and wellbeing, and ensuring the availability of suitable behavioural interventions for all patients.

Evidence from randomized controlled trials suggests that brief advice on smoking in primary care increases rates of smoking cessation relative to minimal or no intervention [4, 9, 10]. The type of advice provided appears to be important. A meta‐analysis of brief opportunistic smoking cessation interventions found that while providing advice to quit on medical grounds increased the rate of quit attempts, offering assistance (either in the form of behavioural or pharmacological support) was significantly more effective in encouraging smokers to attempt to quit [3]. Traditionally, health professionals could offer smokers two effective categories of cessation support: prescription medication (e.g. varenicline) and referral to the stop smoking services, which can provide a combination of behavioural support and prescription medication. Since the National Health Service gave up control of stop smoking services in 2013 budget cuts have seen stop smoking services decommissioned, with only 56% of local authorities now able to offer a universal specialist service in England [11]. In the last decade, the rapid rise in popularity of e‐cigarettes has led many smokers to seek advice on these devices from a health professional [12, 13]. A growing body of research demonstrates the effectiveness of e‐cigarettes as an aid to cessation [14, 15, 16]. In a recent trial conducted in UK stop smoking services, 1‐year abstinence rates were almost twice as high among smokers randomized to use an e‐cigarette than those randomized to use nicotine replacement therapy (18.0 versus 9.9%) [15]. Real‐world evidence shows success rates among smokers who attempt to quit using an e‐cigarette are comparable to the most effective licensed medication (varenicline) and higher than those associated with other commonly used cessation aids (e.g. NRT bought over‐the‐counter) [14]. However, whether health professionals should be recommending e‐cigarettes to smokers remains an issue of some debate in the medical community [17, 18], with clinicians unaware or unconvinced by the evidence on efficacy and concerned by possible long‐term health effects [19, 20]. Several organizations in the United Kingdom, including Public Health England, the Royal College of Physicians and the Royal College of General Practitioners, now recommend that clinicians give advice on e‐cigarettes as one option to help their patients quit smoking [20]. International surveys conducted between 2013 and 2016 have indicated that discussions between smokers and health professionals about nicotine vaping products are infrequent among countries with different regulatory environments (England, Canada, United States, Australia) and only a low percentage of health professionals recommend vaping products [12, 21, 22]. The extent to which smokers are receiving advice on e‐cigarettes in primary care has not been investigated in a representative sample in England.

Despite the strong evidence base on the effectiveness of brief advice on smoking and the requirement for health professionals to encourage healthy behaviour change, not all smoking patients receive brief opportunistic advice in primary care [23]. The majority of primary care physicians acknowledge that providing cessation advice to smoking patients is part of their job, but they are often unenthusiastic about the task [24, 25, 26]. Commonly cited barriers to delivering brief advice on smoking include a lack of time and concern about alienating their patients [27, 28]. Health professionals generally believe that advice to quit is most effective and least irritating if given when a patient presents with a smoking‐related illness [27, 29]. As an example of inconsistent provision of advice, a population‐representative survey of adults in England conducted between 2014 and 2016 found that just under half (48.3%) of smokers who had visited their GP in the last year recalled receiving advice on smoking [23]. Moreover, the odds of receiving advice differed substantially according to various socio‐demographic and behavioural characteristics, with smokers more likely to report receiving advice being older, from the North of England, classified as risky drinkers, more motivated to quit, disabled and from lower occupational social grades [23]. However, the type of advice was not examined, so whether and to what extent those who received advice were offered support as opposed to simple advice to stop smoking, and how this varied according to smokers’ characteristics, is not known.

Identifying characteristics of smokers who are less likely to receive an offer of support is important in drawing health professionals’ attention to any biases (conscious or unconscious) that may influence their decisions on whether to provide advice—and if so, what form that advice takes. In particular, if disadvantaged groups, who tend to be more likely to smoke [2], are less likely to receive effective types of advice and support, redressing this balance is a critical step towards tackling inequalities in health. Establishing whether socio‐economic position affects the likelihood that they try to quit in response to advice or an offer of support could help health professionals to tailor the advice they give to the type most likely to encourage smokers to make a quit attempt.

The present study aimed to address the following research questions:

1. What proportion of smokers report receiving any GP advice on smoking in the last 12 months, and what type of advice/support is received most often?
2. To what extent has the proportion of smokers reporting receiving any GP advice on smoking changed between 2016 and 2019, and how has this differed by type of advice/support?
3. To what extent is the receipt of GP advice on smoking associated with smokers’ socio‐demographic and behavioural characteristics (sex, age, ethnicity, occupational social grade, housing tenure, disability, number of children in the household, level of cigarette addiction, daily cigarette consumption, use of roll‐your‐own tobacco and level of alcohol consumption)?
4. To what extent is having received GP advice on smoking associated with quit attempts and cessation, after adjustment for socio‐demographic and behavioural characteristics?
5. Do any associations between different types of GP advice on smoking and quit attempts or cessation differ according to smokers’ occupational social grade?

## Method

### Design and population

Data were drawn from the ongoing Smoking Toolkit Study (STS), a monthly cross‐sectional survey of representative samples of adults (aged ≥ 16 years) in England designed to provide insights into population‐wide influences on smoking [30]. The study uses a form of random location sampling to select a new sample of approximately 1700 adults aged ≥ 16 years each month. The survey typically covers 200–300 census output areas each wave, which are sampled at random (after stratification by geodemographic analysis of the population) from more than 170 000 adults. Interviewers travel to the selected areas and perform computer‐assisted interviews with one participant aged more than 16 years per household until quotas based upon factors influencing the probability of being at home (working status, age and gender) are fulfilled. Random location sampling is considered superior to conventional quota sampling because the choice of properties approached is reduced by the random allocation of small output areas. However, interviewers can still choose which houses within these areas are most likely to fulfil their quotas, rather than being sent to specific households in advance. Response rates are therefore not appropriate to record, unlike random probability sampling, where interviewers have no choice as to the properties sampled and so response at each address can be recorded. Comparisons suggest that STS estimates for consumption and national sales are closely aligned [31], and national survey data and recorded sales indicate that other key STS variables such as socio‐demographics and smoking prevalence are nationally representative [30, 31].

For the present study, we used aggregated data from respondents to the survey in the period from September 2016 (the first wave to ask smokers who had visited their GP about receipt of advice on e‐cigarettes) to October 2019 (the most recent wave of data available at the time of analysis).

We used data from respondents who reported smoking daily or occasionally in the last 12 months (‘past‐year smokers’) and, for the majority of analyses, having visited their GP practice in the last 12 months.

### Ethics approval and consent to participate

Ethical approval for the STS was granted originally by the UCL Ethics Committee (ID 0498/001). The data are not collected by UCL and are anonymized when received by UCL.

## Measures

### Smoking status

All participants were asked: ‘Which of the following best applies to you? (1) I smoke cigarettes (including hand‐rolled) every day; (2) I smoke cigarettes (including hand‐rolled), but not every day; (3) I do not smoke cigarettes at all, but I do smoke tobacco of some kind (e.g. pipe, cigar or shisha); (4) I have stopped smoking completely in the last year; (5) I stopped smoking completely more than a year ago; (6) I have never been a smoker (i.e. smoked for a year or more).’ Those who reported smoking in the last year (responses 1–4) were considered past‐year smokers.

### Receipt of GP advice on smoking

Past‐year smokers were asked: ‘Has your GP spoken to you about smoking in the past year (i.e. last 12 months)? (1) Yes, he/she suggested that I go to a specialist stop smoking adviser or group; (2) yes, he/she suggested that I see a nurse in the practice; (3) yes, he/she offered me a prescription for Champix, Zyban, a nicotine patch, nicotine gum or another nicotine product; (4) yes, he/she suggested that I use an e‐cigarette; (5) yes, he/she advised me to stop but did not offer anything; (6) yes, he/she asked me about my smoking but did not advise me to stop smoking; (7) no, I have seen my GP in the last year but he/she has not spoken to me about smoking; (8) no, I have not seen my GP in the last year.’ Those who responded ‘yes’ were able to select multiple responses between 1 and 6 to indicate all types of advice they received. Those who responded ‘no’ were able to select only one response option (7 or 8).

For analysis, receipt of advice was coded 1 for those who selected response options 1–5 and 0 for those who selected response options 6 or 7. Receipt of offer of support was coded 1 for those who selected response options 1–4 and 0 for those who selected response options 5–7. Receipt of specific types of advice or offer of support (e.g. suggested e‐cigarette, offered prescription medication, suggested stop smoking services) were coded 1 for those who selected that form of advice and 0 for those who selected all other response options between 5 and 7. Those who responded that they had not seen their GP in the last year (response 8) were excluded from the majority of analyses (see Statistical analysis section for details).

### Socio‐demographic characteristics

Socio‐demographic variables included sex, age, ethnicity, occupational social grade, region, housing tenure, disability and the presence of children in the household. Age was categorized as 16–24, 25–34, 35–44, 45–54, 55–64 and ≥ 65 years. Ethnicity was categorized as white versus non‐white. Occupational social grade was categorized as ABC1 (which includes managerial, professional and intermediate occupations) versus C2DE (which includes small employers and own‐account workers, lower supervisory and technical occupations and semi‐routine and routine occupations, never workers and long‐term unemployed). This measure of social grade is a valid index of socio‐economic position that is widely used in research in UK populations. It has been identified as particularly relevant in the context of tobacco use and quitting [32] and other addictive behaviours [33]. These occupational social grades are frequently amalgamated into two groupings; ABC1 and C2DE. Here, researchers frequently interpret ABC1 to represent people from more and C2DE less advantaged social grades. Housing tenure was categorized as home owner (home owned outright or on a mortgage) versus other (home rented privately, rented from council or housing association or other). We included housing tenure in addition to occupational social grade because it has been identified as a particularly strong predictor of smoking status and health inequalities [34, 35]. Region was categorized as northern, central and southern England according to Government Office Region. Disability status was identified from the question: ‘Do you consider yourself to have a disability within the meaning of the Disability Discrimination Act 1995 (yes/no)?’. The number of children in the household was self‐reported and dichotomized to 0 versus ≥ 1.

### Behavioural characteristics

Behavioural characteristics included level of cigarette addiction, daily cigarette consumption, use of roll‐your‐own tobacco and (because it is strongly associated with smoking) level of alcohol consumption.

Level of cigarette addiction was assessed by self‐reported ratings of the strength of urges to smoke over the last 24 hours [not at all (coded 0), slight (1), moderate (2), strong (3), very strong (4), extremely strong (5)]. This item was also coded 0 for smokers who responded ‘not at all’ to the (separate) question: ‘How much of the time have you spent with the urge to smoke?’ [36]. This measure has been validated and performs at least as well as the Fagerström Test of Cigarette Dependence and the Heaviness of Smoking Index in predicting quitting activity [37], while not being subject to bias due to population‐level changes in cigarette consumption during the study time‐period [31].

Participants were asked to report the number of cigarettes they smoked on an average day (currently for current smokers or when they were smoking for recent ex‐smokers) and how many were roll‐your‐own. We categorized participants into light smokers (smoking on average fewer than five cigarettes per day) and moderate‐to‐heavy smokers (five of more cigarettes per day). Use of roll‐your‐own tobacco was analyzed as predominant use (≥ 50% of total cigarette consumption; a definition used by other studies examining roll‐your‐own cigarette usage [38, 39]).

Alcohol consumption was assessed with the Alcohol Use Disorders Identification Test (AUDIT). High‐risk drinking was defined as a score of 8 or higher [40].

### Quit attempts

Quit attempts made by past‐year smokers was assessed with the question: ‘How many serious attempts to stop smoking have you made in the last 12 months? By serious attempt I mean you decided that you would try to make sure you never smoked again. Please include any attempt that you are currently making, and please include any successful attempt within the last 12 months.’ Those who reported at least one quit attempt were coded 1 and those who reported no quit attempts in the last 12 months were coded 0. Quit attempts referred to attempts to stop smoking combustible tobacco and did not include e‐cigarettes.

### Cessation

Cessation among past‐year smokers was coded 1 for those who answered: ‘I have stopped smoking completely in the last year’ in response to the question assessing current smoking status (response option 4; see full details above under ‘Smoking status’ subheading) and 0 for those who answered that they smoke cigarettes or other tobacco daily or occasionally (response options 1–3). Cessation referred to stopping smoking combustible tobacco and did not include e‐cigarettes.

## Statistical analysis

The analysis plan was pre‐registered on Open Science Framework (https://osf.io/9qnj2/). We made two changes to the analysis following peer review. The first involved changing the coding of the GP advice/offer of support variables to provide a consistent referent category across analyses of associations with quit attempts and cessation. Results using our original (yes/no) coding are available on Open Science Framework for transparency. The second involved analyzing associations of GP advice and offer of support with cessation (in addition to quit attempts, as planned). Analyses were conducted in SPSS version 25 on complete cases. It was not considered appropriate to allow for design clustering, because there was a low ratio of clusters to individuals and complete coverage of England.

We used one‐way independent *t*‐tests and Pearson's χ^2^ tests to test differences in descriptive characteristics between past‐year smokers who did versus did not report having visited their GP in the last 12 months.

### Prevalence estimates

We examined the proportion who reported receiving GP advice on smoking during the last 12 months among (i) all past‐year smokers and (ii) past‐year smokers who reported having visited their GP in the last 12 months. We report the prevalence [and 95% confidence interval (CI)] of receipt of any GP advice, offer of support, each type of support offered and suggestion to use an e‐cigarette, aggregated across the entire study and by quarter. For prevalence estimates, data were weighted to match the English population profile on age, occupational social grade, region, tenure, ethnicity and working status within sex. The dimensions are derived monthly from a combination of the English 2011 census, Office for National Statistics mid‐year estimates and an annual random probability survey conducted for the National Readership Survey. The unequal weighting effect was 1.16, indicating no substantial influence of the sampling weights [41].

### Associations of GP advice and offer of support with socio‐demographic and behavioural characteristics

For the group of respondents who indicated having seen their GP in the last 12 months, we used multivariable logistic regression (on unweighted data) to examine the extent to which receipt of GP advice on smoking was associated with sex, age, ethnicity, occupational social grade, housing tenure, region, disability, children in the household, level of cigarette addiction, daily cigarette consumption, use of roll‐your‐own tobacco and alcohol consumption. Survey year was included as a covariate. There was no substantial multicollinearity.

We constructed five models, with the following variables as binary (yes/no) outcomes: (i) provided any advice on smoking, (ii) offered any support with smoking, (iii) suggested e‐cigarettes, (iv) offered prescription medication and (v) suggested stop smoking services. Results are presented as adjusted odds ratios (ORs) with 95% CIs. To adjust for multiple comparisons, we applied a false discovery rate correction [42] to all *P*‐values using an online calculator (https://www.sdmproject.com/utilities/?show=FDR).

### Associations of GP advice and offer of support with quit attempts and cessation

Associations of receipt of GP advice on smoking (overall and by type of advice) with quit attempts and cessation were analyzed using multivariable logistic regression (on unweighted data), adjusting for the above‐mentioned socio‐demographic and behavioural characteristics and survey year. Among past‐year smokers who reported visiting their GP during the last 12 months, we compared the odds of quit attempts and cessation among those who received any advice (including offer of support), and specifically those who were advised to quit but were not offered any support versus those who did not receive any advice (reference group). Among those who received any advice, we compared the odds of quit attempts and cessation among those who received any offer of support and specific types of support (suggested e‐cigarette, offered prescription medication, suggested stop smoking services, suggested seeing nurse in the practice) versus those who did not receive a specific offer of support.

In order to test whether associations with quit attempts were moderated by social disadvantage, we repeated the models adding the two‐way interactions between receipt of GP advice/support on smoking and occupational social grade. Where there was evidence of moderation, we re‐ran the model in stratified analyses to provide more information as to the nature of the differences between groups.

We calculated Bayes factors [43, 44] to evaluate the strength of evidence for associations with quit attempts and cessation in the magnitude observed in a meta‐analysis of brief opportunistic advice on smoking [3]. The alternative hypothesis was represented by a half‐normal distribution and the expected effect size set to OR = 1.69. Bayes factors ≥ 3 can be interpreted as evidence for the alternative hypothesis (and against the null), ≤ 1/3 as evidence for the null hypothesis and values between 1/3 and 3 suggest that the data are insensitive to distinguish the alternative hypothesis from the null [43, 45].

## Results

### Sample characteristics

A total of 65 123 adults aged ≥ 16 years responded to the Smoking Toolkit Survey between September 2016 and October 2019, 11 588 (17.8%, 95% CI = 17.5–18.1%) of whom were past‐year smokers. Sample characteristics are summarized in Supporting information, Table S1. The majority of past‐year smokers (*n* = 7430; 64.1%, 95% CI = 63.2–65.0%) reported having visited their GP in the last 12 months. Rates of reporting visiting their GP in the last 12 months were significantly higher among smokers who were female, older, from more disadvantaged occupational social grades (C2DE), living in the North of England and disabled (Supporting information, Table S1). They were also higher among moderate/heavy smokers and those who reported stronger urges to smoke, indicating a higher level of cigarette addiction among GP‐visiting smokers (Supporting information, Table S1).

### Prevalence of receipt of GP advice and support on smoking

**FIGURE 1:**
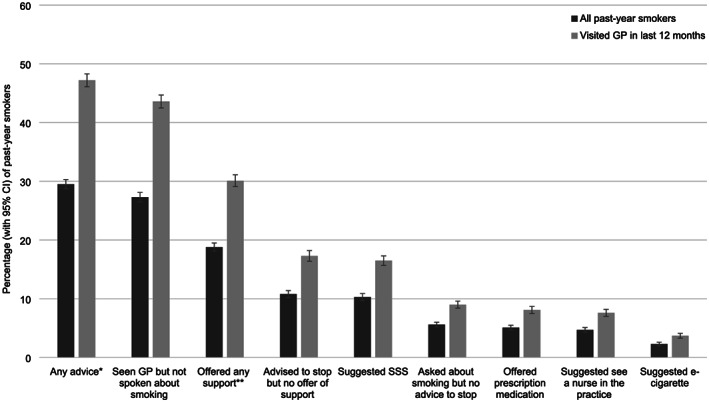
Prevalence of receipt of general practitioner (GP) advice and support on smoking by past‐year smokers in England (2016–19). CI = confidence interval; SSS = stop smoking services

**FIGURE 2:**
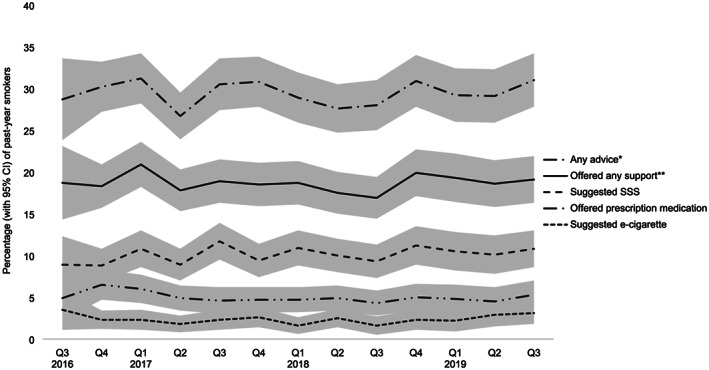
Quarterly prevalence of receipt of general practitioner (GP) advice and support on smoking. CI = confidence interval; SSS = stop smoking services. ^*^Includes suggestions that the patient use an e‐cigarette, go to a specialist stop smoking adviser or group or see a nurse in the practice; offer of prescription medication; or advice to stop smoking without offer of support. ^**^Includes suggestions that the patient use an e‐cigarette, go to a specialist stop smoking adviser or group or see a nurse in the practice; or offer of prescription medication

Figure 1 shows the weighted prevalence of receipt of GP advice and support on smoking aggregated across the entire study period. Of past‐year smokers who reported having visited their GP in the last 12 months, 47.2% [95%CI 46.1‐48.3%] (29.5% [28.7‐30.3%] of all past‐year smokers in England) reported receiving any advice on smoking and 30.1% [29.1‐31.1%] (18.5% [18.1‐19.5%] of all past‐year smokers in England) reported being offered cessation support. The most common form of support offered was stop smoking services (16.5% [15.7‐17.3%]), followed by prescription medication (8.1% [7.5‐8.7%]) and consultation with a nurse in the practice (7.6% [7.0‐8.2%]). Just 3.7% [3.3‐4.1%] reported having been recommended to use an e‐cigarette; 43.6% [42.5‐44.7%] of those who had visited their GP reported not having spoken about smoking at all and a further 9.0% [8.4‐9.6%] said they had been asked about smoking but received no advice to stop. Examination of data aggregated by quarter (Fig. 2) indicated little change in receipt of different types of advice during the study period (this was confirmed in the multivariable logistic regression models which showed no significant association between receipt of GP advice or offer of support and survey year; Table 1). All prevalence estimates (overall and by quarter) are available in tabular form in Supporting information, Table S2.

**TABLE 1:** Socio‐demographic and behavioural correlates of receipt of GP advice or support on smoking.

		Any advice^a^		Offered any support^b^		Suggested e–cigarette		Offered prescription medication		Suggested SSS	
	*N*	% (*n*)	OR_adj_ (95% CI) *P*	% (*n*)	OR_adj_ (95% CI) *P*	% (*n*)	OR_adj_ (95% CI) *P*	% (*n*)	OR_adj_ (95% CI) *P*	% (*n*)	OR_adj_ (95% CI) *P*
Sex											
Male	3246	48.1 (1559)	1 (ref.)	29.7 (964)	1 (ref.)	4.4 (142)	1 (ref.)	9.1 (297)	1 (ref.)	15.8 (513)	1 (ref.)
Female	3531	49.0 (1729)	1.05 (0.95–1.16) 0.511	32.1 (1134)	1.10 (0.98–1.23) 0.174	3.1 (109)	0.75 (0.57–0.97) 0.066	8.4 (298)	0.97 (0.81–1.16) 0.810	18.4 (648)	1.16 (1.01–1.32) 0.067
Age, years											
16–24	994	34.3 (341)	1 (ref.)	18.8 (187)	1 (ref.)	2.8 (28)	1 (ref.)	4.2 (42)	1 (ref.)	10.5 (104)	1 (ref.)
25–34	1280	43.3 (554)	**1.30 (1.08–1.55) 0.015**	28.5 (364)	**1.42 (1.16–1.75) 0.004**	3.5 (45)	1.31 (0.80–2.14) 0.401	6.3 (80)	1.45 (0.98–2.14) 0.126	17.0 (217)	**1.40 (1.08–1.81) 0.028**
35–44	1099	49.3 (541)	**1.57 (1.31–1.89) < 0.001**	33.1 (363)	**1.68 (1.36–2.08) < 0.001**	2.9 (32)	1.04 (0.61–1.76) 0.931	9.7 (107)	**2.25 (1.54–3.28) < 0.001**	18.1 (199)	**1.44 (1.10–1.88) 0.020**
45–54	1209	52.6 (636)	**1.82 (1.52–2.19) < 0.001**	34.9 (421)	**1.88 (1.53–2.31) < 0.001**	4.2 (51)	1.37 (0.84–2.23) 0.321	10.2 (123)	**2.30 (1.59–3.34) < 0.001**	18.6 (225)	**1.58 (1.22–2.05) 0.004**
55–64	1060	57.5 (609)	**2.29 (1.89–2.78) < 0.001**	36.5 (387)	**2.10 (1.70–2.62) < 0.001**	4.4 (47)	1.36 (0.82–2.26) 0.347	10.8 (114)	**2.43 (1.65–3.57) < 0.001**	19.8 (210)	**1.81 (1.38–2.38) < 0.001**
≥ 65	1135	53.5 (607)	**2.09 (1.72–2.55) < 0.001**	33.2 (376)	**1.91 (1.53–2.38) < 0.001**	4.2 (48)	1.32 (0.79–2.22) 0.408	11.4 (129)	**2.73 (1.86–4.03) < 0.001**	18.1 (206)	**1.71 (1.29–2.25) < 0.001**
Ethnicity											
Non–white	689	50.2 (345)	1 (ref.)	34.8 (239)	1 (ref.)	4.5 (31)	1 (ref.)	10.4 (72)	1 (ref.)	19.7 (136)	1 (ref.)
White	6088	48.4 (3288)	**0.80 (0.67–0.94) 0.021**	30.6 (1859)	**0.75 (0.63–0.90) 0.006**	3.6 (220)	0.75 (0.50–1.12) 0.255	8.6 (523)	**0.68 (0.52–0.89) 0.015**	16.8 (1025)	**0.78 (0.63–0.96) 0.043**
Occupational social grade											
ABC1 (advantaged)	2890	46.3 (1338)	1 (ref.)	28.9 (835)	1 (ref.)	3.7 (106)	1 (ref.)	7.5 (217)	1 (ref.)	16.3 (472)	1 (ref.)
C2DE (disadvantaged)	3887	50.2 (1950)	0.98 (0.88–1.08) 0.738	32.5 (1263)	1.01 (0.90–1.13) 0.931	3.7 (145)	1.01 (0.77–1.33) 0.957	9.7 (378)	1.18 (0.98–1.42) 0.165	17.7 (689)	0.96 (0.84–1.10) 0.702
Region											
North	2280	48.5 (1104)	1 (ref.)	28.7 (653)	1 (ref.)	2.8 (63)	1 (ref.)	9.4 (215)	1 (ref.)	15.7 (358)	1 (ref.)
Central	2017	46.3 (934)	0.87 (0.77–0.99) 0.068	29.1 (587)	0.96 (0.84–1.10) 0.702	4.4 (88)	**1.61 (1.15–2.24) 0.015**	8.2 (166)	0.85 (0.68–1.05) 0.215	15.0 (302)	0.89 (0.75–1.05) 0.264
South	2480	50.4 (1250)	1.09 (0.97–1.22) 0.264	34.6 (858)	**1.32 (1.16–1.50) < 0.001**	4.0 (100)	1.45 (1.05–2.01) 0.056	8.6 (214)	0.89 (0.73–1.09) 0.367	20.2 (501)	**1.37 (1.17–1.59) < 0.001**
Home owner											
No	4087	49.6 (2026)	1 (ref.)	31.9 (1304)	1 (ref.)	3.5 (144)	1 (ref.)	8.9 (364)	1 (ref.)	17.7 (724)	1 (ref.)
Yes	2690	46.9 (1262)	**0.83 (0.74–0.92) 0.004**	29.5 (794)	0.87 (0.77–0.98) 0.056	4.0 (107)	1.09 (0.82–1.44) 0.702	8.6 (231)	0.89 (0.73–1.07) 0.333	16.2 (437)	0.90 (0.78–1.04) 0.252
Disability											
No	5269	46.8 (2462)	1 (ref.)	29.9 (1573)	1 (ref.)	3.7 (193)	1 (ref.)	8.3 (435)	1 (ref.)	16.7 (879)	1 (ref.)
Yes	1508	54.9 (826)	1.12 (0.99–1.27) 0.142	34.9 (525)	1.06 (0.93–1.21) 0.501	3.8 (58)	0.96 (0.70–1.31) 0.852	10.6 (160)	1.06 (0.86–1.29) 0.711	18.7 (282)	1.01 (0.86–1.19) 0.931
Children in the household											
0	4702	48.8 (2294)	1 (ref.)	30.4 (1427)	1 (ref.)	4.1 (192)	1 (ref.)	9.3 (435)	1 (ref.)	16.3 (766)	1 (ref.)
≥ 1	2075	47.9 (994)	1.12 (0.99–1.27) 0.127	32.4 (671)	**1.18 (1.03–1.35) 0.036**	2.8 (59)	0.75 (0.54–1.06) 0.182	7.7 (160)	0.93 (0.74–1.16) 0.676	19.0 (395)	**1.28 (1.09–1.51) 0.006**
Strength of urges to smoke	**–**	**–**	**1.24 (1.19–1.30) < 0.001**	**–**	**1.24 (1.18–1.30) < 0.001**	–	1.10 (0.98–1.24) 0.192	**–**	**1.10 (1.02–1.19) 0.035**	–	**1.24 (1.17–1.32) < 0.001**
Daily cigarette consumption											
Light (< 5 CPD)	1988	38.9 (773)	1 (ref.)	22.9 (455)	1 (ref.)	3.0 (60)	1 (ref.)	6.2 (124)	1 (ref.)	12.2 (242)	1 (ref.)
Moderate/heavy (≥ 5 CPD)	4789	52.5 (2515)	**1.35 (1.20–1.52) < 0.001**	34.3 (1643)	**1.41 (1.24–1.61) < 0.001**	4.0 (191)	1.22 (0.88–1.69) 0.341	9.8 (471)	**1.35 (1.08–1.69) 0.021**	19.2 (919)	**1.42 (1.20–1.68) < 0.001**
Roll‐your‐own cigarette use											
No	3565	48.4 (1725)	1 (ref.)	31.3 (1114)	1 (ref.)	3.5 (124)	1 (ref.)	8.8 (312)	1 (ref.)	17.6 (627)	1 (ref.)
Yes	3212	48.7 (1563)	1.03 (0.93–1.14) 0.702	30.7 (984)	1.00 (0.89–1.11) 0.957	4.0 (127)	1.13 (0.87–1.47) 0.501	8.8 (283)	1.04 (0.87–1.24) 0.782	16.6 (534)	0.96 (0.84–110) 0.702
High‐risk drinking											
No	5256	49.5 (2602)	1 (ref.)	32.6 (1713)	1 (ref.)	3.7 (195)	1 (ref.)	9.1 (476)	1 (ref.)	18.1 (951)	1 (ref.)
Yes	1521	45.2 (686)	0.97 (0.86–1.10) 0.728	25.3 (385)	**0.81 (0.70–0.93) 0.006**	3.7 (56)	1.01 (0.73–1.38) 0.968	7.8 (119)	0.99 (0.79–1.23) 0.931	13.8 (210)	0.83 (0.70–0.98) 0.061
Survey year	–	–	1.02 (0.97–1.08) 0.501	–	1.01 (0.95–1.07) 0.852	–	1.02 (0.89–1.16) 0.874	–	0.97 (0.88–1.06) 0.573	–	1.05 (0.98–1.12) 0.241

Each analysis was conducted on all past‐year smokers who reported having visited their general practitioner (GP) in the last 12 months and who had complete data on all variables in the table. Each column represents a separate multivariable analysis that included all characteristics listed in the left‐hand column. Statistically significant (*P* < 0.05) associations are shown in bold type.

CI = confidence interval; OR = odds ratio; SSS = stop smoking services; CPD = cigarettes per day.

^a^ Includes suggestions that the patient use an e‐cigarette, go to a specialist stop smoking adviser or group or see a nurse in the practice; offer of prescription medication; or advice to stop smoking without offer of support.

^b^ Includes suggestions that the patient use an e‐cigarette, go to a specialist stop smoking adviser or group, or see a nurse in the practice; or offer of prescription medication.

### Associations of GP advice and support with socio‐demographic and behavioural characteristics

Table 1 summarizes multivariable associations between socio‐demographic and behavioural characteristics and receipt of GP advice or support on smoking.

There were no significant sex differences in receipt of advice or support. The odds of receiving any advice, any offer of support and, specifically, offer of prescription medication or suggestion to use stop smoking services were significantly higher among older compared with younger smokers (with the highest odds in those aged 55–64 years) and lower among white compared with non‐white smokers. The odds of advice to use an e‐cigarette, however, did not differ significantly by age or ethnicity. Receipt of advice or support did not differ significantly by occupational social grade, but the odds of being offered any advice were significantly lower among home‐owners than smokers in other housing tenures. There were some regional differences in receipt of advice or support, with significantly higher odds of being recommended an e‐cigarette in central versus northern England and higher odds of being offered any support, and stop smoking services specifically, in the South compared with the North. Disability was not significantly associated with receipt of advice or support, but smokers who had children in the home had significantly higher odds of being offered any support and, specifically, stop smoking services.

Smokers who reported stronger urges to smoke and moderate/heavy cigarette consumption (five or more cigarettes per day) had significantly higher odds of receiving any advice, any offer of support, offer of prescription medication and suggestion to use stop smoking services than those with weaker urges or who smoked less heavily, although odds of receiving advice on e‐cigarettes did not differ significantly. The type of cigarettes smoked was not significantly associated with receipt of advice or support. High‐risk drinkers had significantly lower odds of being offered any support, although specific types of support analyzed did not differ significantly by alcohol consumption. There were no significant differences by survey year.

### Associations of GP advice and support with quit attempts and cessation

A third (34.3%) of past‐year smokers who reported visiting their GP in the last 12 months reported having made at least one serious quit attempt in the past year. Table 2 summarizes associations between receipt of different forms of GP advice or support on smoking and past‐year quit attempts. Overall, the data provided strong evidence that smokers who were offered any advice or offer of support were significantly more likely to report a quit attempt than those who were not. Examination of specific response options indicated that offer of support was important: those who were offered prescription medication or recommended stop smoking services, consultation with a nurse in the practice or e‐cigarettes were significantly more likely to report having made an attempt to quit smoking than those who were not. Associations with suggesting e‐cigarettes and offering prescription medication did not differ significantly by occupational social grade (Supporting information, Table S3). However, associations between offer of any support, suggesting stop smoking services and suggesting seeing a practice nurse and quit attempts were moderated by occupational social grade (Supporting information, Table S3). The data provided moderate evidence that smokers from social grades ABC1 (the more advantaged socio‐economic groups) had significantly higher odds of making a quit attempt if they were advised to stop even in the absence of offer of support, but there was strong evidence that advice to stop without an offer of support was not associated higher odds of making a quit attempt among smokers who were more disadvantaged (Table 2).

**TABLE 2:** Associations between receipt of GP advice or support on smoking and quit attempts.

		OR_adj_^a^	95% CI	*P*	BF	Interpretation of BF
Visited GP in last 12 months						
No advice		1 (ref.)	–	–	–	–
Any advice^a^		**1.95**	**1.75–2.17**	**< 0.001**	> 100	Extremely strong evidence for H1
Advice to stop with no offer of support		1.08	0.95–1.24	0.239	0.44	Data are insensitive
Social grade ABC1		**1.34**	**1.10–1.64**	**0.004**	3.73	Moderate evidence for H1
Social grade C2DE		0.90	0.75–1.08	0.267	0.06	Strong evidence for H0
Received any advice						
No offer of support		1 (ref.)	–	–	–	–
Offered any support^b^		**1.52**	**1.30–1.76**	**< 0.001**	> 100	Extremely strong evidence for H1
Social grade ABC1		1.21	0.96–1.53	0.110	1.02	Data are insensitive
Social grade C2DE		**1.81**	**1.48–2.21**	**< 0.001**	48.25	Very strong evidence for H1
Suggested e‐cigarette		**1.80**	**1.35–2.41**	**< 0.001**	5.88	Moderate evidence for H1
Offered prescription medication		**2.52**	**2.04–3.12**	**< 0.001**	62.77	Very strong evidence for H1
Suggested SSS		**1.39**	**1.17–1.66**	**< 0.001**	9.94	Moderate evidence for H1
Social grade ABC1		1.08	0.82–1.42	0.574	0.43	Data are insensitive
Social grade C2DE		**1.68**	**1.33–2.13**	**< 0.001**	13.52	Strong evidence for H1
Suggested see a nurse in the practice		**1.44**	**1.16–1.80**	**0.001**	5.82	Moderate evidence for H1
Social grade ABC1		1.07	0.76–1.51	0.708	0.46	Data are insensitive
Social grade C2DE		**1.88**	**1.40–2.53**	**< 0.001**	5.73	Moderate evidence for H1

Analyses were conducted on past‐year smokers who reported having visited their general practitioner (GP) in the last 12 months and who had complete data on all covariates. Each form of advice or support was modelled separately. Statistically significant (*P* < 0.05) associations are shown in bold type.

Occupational social grades ABC1 represent more advantaged groups; C2DE more disadvantaged groups.

BF = Bayes factor; CI = confidence interval; H0 = null hypothesis (i.e. advice/support not associated with increased odds of quit attempt); H1 = experimental hypothesis (i.e. advice/support associated with increased odds of quit attempt); OR = odds ratio; SSS = stop smoking services.

^a^ Includes suggestions that the patient use an e‐cigarette, go to a specialist stop smoking adviser or group or see a nurse in the practice; offer of prescription medication; or advice to stop smoking without offer of support.

^b^ Includes suggestions that the patient use an e‐cigarette, go to a specialist stop smoking adviser or group or see a nurse in the practice; or offer of prescription medication.

^c^ Adjusted for sex, age, ethnicity, occupational social grade, region, housing tenure, disability, children in the household, level of cigarette addiction, daily cigarette consumption, use of roll‐your‐own tobacco, alcohol consumption and survey year.

One in 20 (5.4%) past‐year smokers who reported visiting heir GP in the last 12 months reported having quit smoking completely during the last year and were abstinent at the time of the survey. Table 3 summarizes associations between receipt of different forms of GP advice or support on smoking and cessation. Because of the low cessation prevalence, the data were largely insensitive to detect significant differences by type of advice/support offered. Overall, only the offer of prescription medication was significantly associated with increased odds of cessation. However, there was evidence of moderation of associations with offer of any support, suggesting e‐cigarettes, offering prescription medication and seeing a practice nurse by occupational social grade (Supporting information, Table S4), with these associated with increased odds of smoking cessation only in more disadvantaged smokers (social grades C2DE) (Table 3). Similar to the results on quit attempts, the data could not rule out a positive association between advice to stop without an offer of support and cessation among more advantaged smokers (ABC1), but provided strong evidence that advice to stop without an offer of support was not associated with higher odds of cessation among disadvantaged smokers (Table 3).

**TABLE 3:** Associations between receipt of GP advice or support on smoking and cessation.

		OR_adj_^a^	95% CI	*P*	BF	Interpretation of BF
Visited GP in last 12 months						
No advice		1 (ref.)	–	–	–	–
Any advice^a^		1.10	0.87–1.37	0.432	0.49	Data are insensitive
Advice to stop with no offer of support		1.00	0.74–1.34	0.861	0.27	Moderate evidence for H0
Social grade ABC1		1.39	0.95–2.05	0.092	1.37	Data are insensitive
Social grade C2DE		0.67	0.41–1.07	0.095	0.09	Strong evidence for H0
Received any advice						
No offer of support		1 (ref.)	–	–	–	–
Offered any support^b^		1.05	0.75–1.48	0.775	0.42	Data are insensitive
Social grade ABC1		0.71	0.45–1.14	0.158	0.12	Moderate evidence for H0
Social grade C2DE		**1.74**	**1.02–2.98**	**0.043**	1.49	Data are insensitive
Suggested e‐cigarette		0.79	0.39–1.63	0.526	0.33	Data are insensitive
Social grade ABC1		**0.22**	**0.06–0.84**	**0.026**	0.04	Strong evidence for H0
Social grade C2DE		2.34	0.90–6.07	0.080	1.13	Data are insensitive
Offered prescription medication		**1.73**	**1.13–2.66**	**0.012**	1.97	Data are insensitive
Social grade ABC1		1.06	0.56–2.06	0.842	0.65	Data are insensitive
Social grade C2DE		**2.80**	**1.51–5.20**	**0.001**	1.41	Data are insensitive
Suggested SSS		0.88	0.59–1.33	0.555	0.22	Moderate evidence for H0
Suggested see a nurse in the practice		1.01	0.61–1.69	0.964	0.48	Data are insensitive
Social grade ABC1		0.53	0.24–1.18	0.120	0.12	Moderate evidence for H0
Social grade C2DE		2.11	1.00–4.46	0.051	1.26	Data are insensitive

Analyses were conducted on past‐year smokers who reported having visited their general practitioner (GP) in the last 12 months and who had complete data on all covariates. Each form of advice or support was modelled separately. Statistically significant (*P* < 0.05) associations are shown in bold type.

Occupational social grades ABC1 represent more advantaged groups; C2DE more disadvantaged groups.

BF = Bayes factor; CI = confidence interval; H0 = null hypothesis (i.e. advice/support not associated with increased odds of quit attempt); H1 = experimental hypothesis (i.e. advice/support associated with increased odds of quit attempt); OR = odds ratio; SSS = stop smoking services.

^a^ Includes suggestions that the patient use an e‐cigarette, go to a specialist stop smoking adviser or group or see a nurse in the practice; offer of prescription medication; or advice to stop smoking without offer of support.

^b^ Includes suggestions that the patient use an e‐cigarette, go to a specialist stop smoking adviser or group or see a nurse in the practice; or offer of prescription medication.

^c^ Adjusted for sex, age, ethnicity, occupational social grade, region, housing tenure, disability, children in the household, level of cigarette addiction, daily cigarette consumption, use of roll‐your‐own tobacco, alcohol consumption and survey year.

## Discussion

In this large, representative survey of smokers in England, one in two past‐year smokers who reported having visited their GP in the last 12 months recalled receiving any advice on smoking and one in three reported being offered cessation support. One in six smokers reported being offered support from stop smoking services, one in 12 was offered prescription medication and one in 27 smokers recalled advice to use an e‐cigarette. Smokers who were older, non‐white, more addicted and who smoked five or more cigarettes per day had consistently higher odds of reporting receiving advice or offer of support. There were some differences by region, housing tenure, presence of children in the home and high‐risk drinking in the types of advice/support received. There were no significant differences by sex, occupational social grade, disability, type of cigarettes smoked or survey year. Advice with any offer of support was associated with higher odds of reporting an attempt to quit independently of socio‐economic position, relative to advice alone. Advice alone was associated with higher odds of quit attempts in smokers from higher but not lower occupational social grades, relative to no advice. Advice with offer of support was associated with higher odds of cessation in smokers from lower but not higher occupational social grades, relative to advice alone. Advice alone was not associated with higher odds of cessation in smokers from lower occupational social grades, relative to no advice, but data were inconclusive for higher social grade smokers.

The prevalence of recall of receipt of advice on smoking by smokers in England who visited their GP, at just under 50% between 2016 and 2019, is consistent with medical records and patient surveys between 2000 and 2009 [46, 47] and data collected as part of the Smoking Toolkit Study between 2014 and 2016 [5, 23]. The relatively lower rate of offer of support (~30%) suggests that even GPs who offer advice on smoking may be missing an opportunity to boost quitting rates, given evidence that offering cessation support is significantly more effective than advice alone in encouraging smokers to make a quit attempt [3] (a finding supported by the present results). Analysis of the type of support offered provided interesting insights: most common were stop smoking services and prescription medication, both of which have been demonstrated to be effective in helping smokers to quit [48, 49, 50]. However, in line with previous research [12, 21, 22], very few GPs appeared to have recommended the use of e‐cigarettes despite these being the most popular quitting aid used by smokers in England [51] and growing evidence of their effectiveness [14, 15, 16] and cost‐effectiveness [52]. A recent survey of UK GPs and practice nurses indicated that many are reluctant to recommend e‐cigarettes to their patients because they lack knowledge on the products, and are concerned about their long‐term safety [20]. However, e‐cigarettes are frequently raised in conversations with patients who smoke, and the majority of clinicians said they would like more training on e‐cigarettes [20].

The low prevalence of receipt of advice with an offer of support did not change during the study period, suggesting no recent progress in persuading more GPs to offer assistance. During this period, there have been sustained real cuts to health‐care and public health, including spending on tobacco control [11]. Despite efforts by medical and public health organizations for GPs to provide advice on e‐cigarettes, there was a consistently low prevalence of e‐cigarettes being suggested throughout the study period. In 2016, a landmark report by the Royal College of Physicians recommended that: **‘**in the interests of public health it is important to promote the use of e‐cigarettes, NRT and other non‐tobacco nicotine products as widely as possible as a substitute for smoking in the UK’ [53]. In 2017, the Royal College of General Practitioners issued a position statement on e‐cigarettes which made several recommendations on advice primary‐care clinicians should offer to smokers, including that they should: (i) advise smokers that behavioural support and prescription medication from local stop smoking services is the most effective quit method; (ii) provide referral to stop smoking services where these services exist, and the patient wishes to access this support; and (iii) use their clinical judgement on an individual patient basis and consider promoting e‐cigarette use as a means to stopping smoking combustible tobacco [54].

Smokers who were more addicted were more likely to receive advice or an offer of support. It is possible that this was because their smoking status was observable to their GP; for example, because they had a documented smoking‐related disease. Also, smokers who are more addicted are often more likely to seek support with quitting, and this enthusiasm may have been detected by GPs [55, 56, 57].

In contrast with previous studies that have documented differences in recall of GP advice on smoking among smokers by occupational social grade and education [5, 23], we did not observe significant socio‐economic inequalities in recall of advice or offer of support. There were no significant differences by occupational social grade, but while offer of support (overall and by type of support) did not differ by housing tenure, smokers who did not own their own homes were slightly more likely to recall receiving advice on smoking. Ensuring that disadvantaged smokers are offered cessation support is particularly important, given that they tend to be more addicted and less likely to be successful in quitting despite being at least as motivated to do so as more affluent smokers [58, 59]. Our finding that advice to stop smoking without an offer of support was associated with increased quitting activity in more advantaged but not less advantaged smokers emphasizes this disparity and the need to offer support to disadvantaged smokers.

A key strength of the study is the large, representative sample. However, there were also limitations. First, the data relied upon self‐reports of receipt of GP advice on smoking, introducing scope for recall bias that may affect estimates of prevalence. In addition, if smokers with certain socio‐demographic or behavioural characteristics or those who did not report a quit attempt were less likely to recall advice they have received, this may have biased estimates of associations with these variables. Secondly, data were only collected on receipt of advice from a GP, so we were not able to explore advice received from other health professionals. All health professionals in England are encouraged to ‘make every contact count’ and use routine interactions with patients to engage in opportunistic conversations about improving their health by addressing risk factors such as smoking [60]. Finally, the cross‐sectional study design limited our ability to establish a causal relationship between advice on smoking and quit attempts. Receipt of advice on smoking and quit attempts were reported retrospectively during the same time‐period, so we were not able to determine which came first: the advice or the quit attempt. Prevalence estimates may underestimate the provision of advice or offer of support if a substantial proportion of participants quit smoking before visiting their GP in the 12‐month recall period. Unsuccessful quit attempts may have led participants to ask their GP for advice.

## Conclusions

In England, a minority (30%) of smokers who visit their GP report receiving an offer of support for smoking cessation. Smokers’ characteristics are related to receiving advice or offer of support: notably, those most likely to receive advice or support are older, from ethnic minority groups, smoke more heavily, without housing tenure and are more addicted. While provision of simple advice to quit may be sufficient to motivate more socio‐economically advantaged smokers to make a quit attempt, offering pharmacological and/or behavioural support appears to be important in encouraging less advantaged smokers to quit and thus reducing smoking‐related health inequalities.

## Declaration of interests

J.B. has received unrestricted research funding from Pfizer, who manufacture smoking cessation medications. All authors declare no financial links with tobacco companies or e‐cigarette manufacturers or their representatives.

## Author Contributions

**Sarah Jackson:** Conceptualization; formal analysis; investigation; methodology; visualization. **Claire Garnett:** Conceptualization; investigation; methodology. **Jamie Brown:** Conceptualization; data curation; funding acquisition; investigation; methodology; resources; supervision.

## Supporting information

**Table S1** Sociodemographic and behavioural characteristics of all past‐year smokers and those who reported having visited their GP in the last 12 months
**Table S2** Weighted prevalence of receipt of GP advice on smoking, overall and by quarter
**Table S3** Interactions between receipt of GP advice or support on smoking and social grade on quit attempts
**Table S4** Interactions between receipt of GP advice or support on smoking and social grade on cessation.
