# Supplementary material for: Prevalence and correlates of receipt by smokers of general practitioner advice on smoking cessation in England: a cross‐sectional survey of adults
Source: Addiction. 2020 Jul 24;116(2):358–72. doi: 10.1111/add.15187 (PMC8432152; doi:10.1111/add.15187)
Supplement: Supplementary file 1 — Table S1 Sociodemographic and behavioural characteristics of all past‐year smokers and those who reported having visited their GP in the last 12 months Table S2 Weighted prevalence of receipt of GP advice on smoking, overall and by quarter Table S3 Interactions between receipt of GP advice or support on smoking and social grade on quit attempts Table S4 Interactions between receipt of GP advice or support on smoking and social grade on cessation. [file ADD-116-358-s001.docx]

| **Supplementary Table 1** Sociodemographic and behavioural characteristics of all past-year smokers and those who reported having visited their GP in the last 12 months | | | | |
| --- | --- | --- | --- | --- |
|  | | **All past-year smokers,**  ***n*** | **Visited GP in last 12 months,**  **% (*n*)** | ***P*^1^** |
| Total | | 11,588 | 64.1 (7430) | - |
| Sex | |  |  | <0.001 |
|  | Male | 6118 | 59.3 (3629) | - |
|  | Female | 5470 | 69.5 (3801) | - |
| Age in years | |  |  |  |
|  | 16-24 | 2041 | 53.9 (1100) | <0.001 |
|  | 25-34 | 2321 | 59.5 (1382) | - |
|  | 35-44 | 1858 | 63.1 (1172) | - |
|  | 45-54 | 1970 | 66.8 (1316) | - |
|  | 55-64 | 1692 | 68.5 (1159) | - |
|  | ≥65 | 1706 | 76.3 (1301) | - |
| Ethnicity | |  |  | 0.218 |
|  | Non-white | 1271 | 62.5 (795) | - |
|  | White | 10273 | 64.3 (6606) | - |
| Social grade | |  |  | 0.002 |
|  | ABC1 (advantaged) | 5107 | 62.6 (3195) | - |
|  | C2DE (disadvantaged) | 6481 | 65.3 (4235) | - |
| Region | |  |  |  |
|  | North | 3726 | 65.4 (2436) | 0.022 |
|  | Central | 3500 | 62.3 (2182) | - |
|  | South | 4362 | 64.5 (2812) | - |
| Home owner | |  |  | 0.573 |
|  | No | 6867 | 64.4 (4419) | - |
|  | Yes | 4629 | 63.8 (2955) | - |
| Disability | |  |  | <0.001 |
|  | No | 9521 | 60.5 (5758) | - |
|  | Yes | 2023 | 81.5 (1649) | - |
| Children in the household | |  |  | 0.237 |
|  | 0 | 8049 | 64.5 (5189) | - |
|  | ≥1 | 3539 | 63.3 (2241) | - |
| Strength of urges to smoke (0-5), mean (SD) | | 1.77 (1.15) | 1.84 (1.17) | <0.001 |
| Daily cigarette consumption | |  |  | 0.001 |
|  | Light (<5 CPD) | 3386 | 61.9 (2096) | - |
|  | Moderate/heavy (≥5 CPD) | 7654 | 65.3 (4996) | - |
| Roll-your-own cigarette use | |  |  | 0.264 |
|  | No | 5646 | 65.1 (3677) | - |
|  | Yes | 5150 | 64.1 (3301) | - |
| High-risk drinking | |  |  | 0.414 |
|  | No | 8829 | 64.2 (5670) | - |
|  | Yes | 2611 | 63.3 (1654) | - |
| CPD; cigarettes per day. GP; general practitioner. SD; standard deviation.  *Note*: there were some missing data for ethnicity (*n*=44), housing tenure (*n*=92), disability (*n*=44), strength of urges to smoke (*n*=45), daily cigarette consumption (*n*=548), roll-your-own cigarette use (*n*=792), and high-risk drinking (*n*=148). Valid percentages are shown for ease of interpretation.  ^1^ *p* values are for the difference in rates of reporting having visited their GP in the last 12 months. | | | | |

| **Supplementary Table 2** Weighted prevalence of receipt of GP advice on smoking, overall and by quarter | | | | | | | | | | |
| --- | --- | --- | --- | --- | --- | --- | --- | --- | --- | --- |
| **% (*n*)** |  | Any advice* | Offered any support** | Suggested e-cigarette | Offered prescription medication | Suggested SSS | Suggested see a nurse | Advised to stop but no offer of support | Asked about smoking but no advice to stop | Seen GP but not spoken about smoking |
| **All past-year smokers** | |  |  |  |  |  |  |  |  |  |
| Overall |  | 29.5 (3530) | 18.8 (2250) | 2.3 (275) | 5.1 (605) | 10.3 (1232) | 4.7 (566) | 10.8 (1296) | 5.6 (670) | 27.3 (3261) |
| *Year* | *Quarter* |  |  |  |  |  |  |  |  |  |
| 2016 | Q3 | 28.7 (106) | 18.7 (69) | 3.5 (13) | 4.9 (18) | 8.9 (33) | 5.7 (21) | 10.0 (37) | 5.9 (22) | 28.4 (105) |
|  | Q4 | 30.2 (291) | 18.3 (176) | 2.3 (22) | 6.5 (63) | 8.8 (85) | 5.4 (52) | 12.5 (120) | 9.2 (89) | 26.0 (250) |
| 2017 | Q1 | 31.2 (293) | 20.9 (196) | 2.3 (22) | 6.0 (56) | 10.8 (102) | 6.2 (58) | 10.6 (100) | 6.2 (58) | 28.4 (267) |
|  | Q2 | 26.7 (272) | 17.8 (182) | 1.8 (18) | 4.9 (50) | 8.9 (91) | 5.0 (51) | 8.9 (91) | 7.2 (74) | 27.7 (283) |
|  | Q3 | 30.5 (289) | 18.9 (179) | 2.3 (22) | 4.6 (44) | 11.7 (111) | 3.7 (35) | 11.6 (110) | 5.9 (56) | 28.5 (270) |
|  | Q4 | 30.8 (294) | 18.5 (177) | 2.6 (25) | 4.7 (45) | 9.4 (90) | 5.2 (50) | 12.3 (118) | 4.2 (40) | 28.2 (270) |
| 2018 | Q1 | 28.9 (288) | 18.7 (186) | 1.6 (16) | 4.7 (47) | 10.9 (109) | 4.4 (44) | 10.1 (101) | 4.7 (47) | 28.4 (283) |
|  | Q2 | 27.6 (278) | 17.5 (176) | 2.5 (25) | 4.9 (49) | 10.0 (101) | 4.8 (48) | 10.1 (102) | 5.6 (56) | 27.2 (274) |
|  | Q3 | 28.0 (272) | 16.9 (164) | 1.6 (16) | 4.3 (42) | 9.3 (91) | 3.6 (35) | 11.2 (109) | 3.8 (37) | 28.7 (280) |
|  | Q4 | 30.9 (283) | 19.9 (182) | 2.3 (21) | 5.0 (46) | 11.2 (103) | 6.2 (57) | 11.0 (101) | 4.6 (42) | 26.1 (239) |
| 2019 | Q1 | 29.2 (247) | 19.3 (164) | 2.2 (19) | 4.8 (41) | 10.5 (89) | 4.5 (38) | 10.0 (85) | 5.1 (43) | 26.9 (228) |
|  | Q2 | 29.1 (250) | 18.6 (18.6) | 2.9 (25) | 4.5 (39) | 10.1 (87) | 4.2 (36) | 10.6 (91) | 5.1 (44) | 26.8 (230) |
|  | Q3 | 31.0 (272) | 19.1 (168) | 3.1 (27) | 5.3 (47) | 10.8 (95) | 3.5 (31) | 11.9 (105) | 5.6 (49) | 23.8 (209) |
|  | Q4 | 34.4 (95) | 25.5 (70) | 1.5 (4) | 6.5 (18) | 16.0 (44) | 3.6 (10) | 8.7 (24) | 5.1 (14) | 26.5 (73) |
| **Visited GP in last 12 months** | |  |  |  |  |  |  |  |  |  |
| Overall |  | 47.2 (3530) | 30.1 (2250) | 3.7 (275) | 8.1 (605) | 16.5 (1232) | 7.6 (566) | 17.3 (1296) | 9.0 (670) | 43.6 (3261) |
| *Year* | *Quarter* |  |  |  |  |  |  |  |  |  |
| 2016 | Q3 | 45.7 (106) | 29.6 (69) | 5.6 (13) | 7.7 (18) | 14.2 (33) | 9.0 (21) | 15.9 (37) | 9.4 (22) | 45.1 (105) |
|  | Q4 | 46.1 (291) | 27.9 (176) | 3.5 (22) | 10.0 (63) | 13.5 (85) | 8.2 (52) | 19.0 (120) | 14.1 (89) | 39.6 (250) |
| 2017 | Q1 | 47.4 (293) | 31.7 (196) | 3.5 (22) | 9.0 (56) | 16.5 (102) | 9.4 (58) | 16.2 (100) | 9.4 (58) | 43.1 (267) |
|  | Q2 | 43.2 (272) | 28.9 (182) | 2.9 (18) | 7.9 (50) | 14.4 (91) | 8.1 (51) | 14.4 (91) | 11.7 (74) | 44.9 (283) |
|  | Q3 | 47.0 (289) | 29.1 (179) | 3.6 (22) | 7.2 (44) | 18.0 (111) | 5.7 (35) | 17.9 (110) | 9.1 (56) | 43.9 (270) |
|  | Q4 | 48.7 (294) | 29.4 (177) | 4.1 (25) | 7.4 (45) | 14.8 (90) | 8.2 (50) | 19.4 (118) | 6.6 (40) | 44.5 (270) |
| 2018 | Q1 | 46.6 (288) | 30.1 (186) | 2.6 (16) | 7.6 (47) | 17.6 (109) | 7.1 (44) | 16.3 (101) | 7.6 (47) | 45.6 (283) |
|  | Q2 | 45.7 (278) | 28.9 (176) | 4.1 (25) | 8.0 (49) | 16.5 (101) | 7.9 (48) | 16.7 (102) | 9.2 (56) | 44.9 (274) |
|  | Q3 | 46.2 (272) | 27.8 (164) | 2.7 (16) | 7.1 (42) | 15.4 (91) | 5.9 (35) | 18.4 (109) | 6.3 (37) | 47.3 (280) |
|  | Q4 | 50.2 (283) | 32.3 (182) | 3.7 (21) | 8.1 (46) | 18.2 (103) | 10.1 (57) | 17.9 (101) | 7.4 (42) | 42.3 (239) |
| 2019 | Q1 | 47.7 (247) | 31.6 (164) | 3.7 (19) | 7.9 (41) | 17.1 (89) | 7.3 (38) | 16.4 (85) | 8.3 (43) | 43.9 (228) |
|  | Q2 | 47.7 (250) | 30.6 (160) | 4.8 (25) | 7.5 (39) | 16.6 (87) | 6.9 (36) | 17.4 (91) | 8.4 (44) | 44.0 (230) |
|  | Q3 | 51.3 (272) | 31.7 (168) | 5.1 (27) | 8.9 (47) | 17.9 (95) | 5.8 (31) | 19.8 (105) | 9.2 (49) | 39.3 (209) |
|  | Q4 | 52.2 (95) | 38.5 (70) | 2.2 (4) | 9.9 (18) | 24.2 (44) | 5.5 (10) | 13.2 (24) | 7.7 (14) | 40.1 (73) |
| ^1^ Includes suggestions that the patient use an e-cigarette, go to a specialist stop smoking advisor or group, or see a nurse in the practice; offer of prescription medication; or advice to stop smoking without offer of support.  ^2^ Includes suggestions that the patient use an e-cigarette, go to a specialist stop smoking advisor or group, or see a nurse in the practice; or offer of prescription medication. | | | | | | | | | | |

| **Supplementary Table 3** Interactions between receipt of GP advice or support on smoking and social grade on quit attempts | | | |  |
| --- | --- | --- | --- | --- |
|  | **Interaction with social grade** | | | |
|  | **OR_adj_^1^** | **95% CI** | ***p*** | |
| Any advice^2^ | 0.99 | 0.80-1.22 | 0.900 | |
| Advised to stop but no offer of support | 0.67 | 0.51-0.87 | 0.003 | |
| Offered any support^3^ | 1.52 | 1.12-2.06 | 0.007 | |
| Suggested e-cigarette | 1.52 | 0.85-2.73 | 0.160 | |
| Offered prescription medication | 1.43 | 0.93-2.20 | 0.106 | |
| Suggested SSS | 1.54 | 1.09-2.18 | 0.016 | |
| Suggested see a nurse in the practice | 1.86 | 1.20-2.90 | 0.006 | |
| CI; confidence interval. GP; general practitioner. OR; odds ratio. SSS; stop smoking services.  ^1^ Adjusted for sex, age, ethnicity, social grade, region, housing tenure, disability, children in the household, level of cigarette addiction, daily cigarette consumption, use of roll-your-own tobacco, alcohol consumption, and survey year.  ^2^ Includes suggestions that the patient use an e-cigarette, go to a specialist stop smoking advisor or group, or see a nurse in the practice; offer of prescription medication; or advice to stop smoking without offer of support.  ^3^ Includes suggestions that the patient use an e-cigarette, go to a specialist stop smoking advisor or group, or see a nurse in the practice; or offer of prescription medication. | | | |  |

| **Supplementary Table 4** Interactions between receipt of GP advice or support on smoking and social grade on cessation | | | |  |
| --- | --- | --- | --- | --- |
|  | **Interaction with social grade** | | | |
|  | **OR_adj_^1^** | **95% CI** | ***p*** | |
| Any advice^2^ | 0.79 | 0.51-1.23 | 0.294 | |
| Advised to stop but no offer of support | 0.48 | 0.26-0.89 | 0.019 | |
| Offered any support^3^ | 2.38 | 1.18-4.80 | 0.016 | |
| Suggested e-cigarette | 10.22 | 2.13-49.10 | 0.004 | |
| Offered prescription medication | 2.51 | 1.04-6.06 | 0.040 | |
| Suggested SSS | 1.79 | 0.79-4.08 | 0.166 | |
| Suggested see a nurse in the practice | 4.12 | 1.43-11.91 | 0.009 | |
| CI; confidence interval. GP; general practitioner. OR; odds ratio. SSS; stop smoking services.  ^1^ Adjusted for sex, age, ethnicity, social grade, region, housing tenure, disability, children in the household, level of cigarette addiction, daily cigarette consumption, use of roll-your-own tobacco, alcohol consumption, and survey year.  ^2^ Includes suggestions that the patient use an e-cigarette, go to a specialist stop smoking advisor or group, or see a nurse in the practice; offer of prescription medication; or advice to stop smoking without offer of support.  ^3^ Includes suggestions that the patient use an e-cigarette, go to a specialist stop smoking advisor or group, or see a nurse in the practice; or offer of prescription medication. | | | |  |
